# An Experimental Insight into Extracellular Phosphatases – Differential Induction of Cell-Specific Activity in Green Algae Cultured under Various Phosphorus Conditions

**DOI:** 10.3389/fmicb.2018.00271

**Published:** 2018-02-21

**Authors:** Jaroslav Vrba, Markéta Macholdová, Linda Nedbalová, Jiří Nedoma, Michal Šorf

**Affiliations:** ^1^Department of Ecosystem Biology, Faculty of Science, University of South Bohemia, České Budějovice, Czechia; ^2^Institute of Hydrobiology, Biology Centre CAS, České Budějovice, Czechia; ^3^Department of Ecology, Faculty of Science, Charles University, Prague, Czechia; ^4^Department of Zoology, Fisheries, Hydrobiology and Apiculture, Faculty of AgriSciences, Mendel University, Brno, Czechia

**Keywords:** acid phosphatase, *Coccomyxa*, ELF97 phosphate, FLEA technique, image cytometry, inorganic phosphorus, organic phosphorus, phosphorus limitation

## Abstract

Extracellular phosphatase activity (PA) has been used as an overall indicator of P depletion in lake phytoplankton. However, detailed insights into the mechanisms of PA regulation are still limited, especially in the case of acid phosphatases. The novel substrate ELF97 phosphate allows for tagging PA on single cells in an epifluorescence microscope. This fluorescence-labeled enzyme activity (FLEA) assay enables for autecological studies in natural phytoplankton and algal cultures. We combined the FLEA assay with image analysis to measure cell-specific acid PA in two closely related species of the genus *Coccomyxa* (Trebouxiophyceae, Chlorophyta) isolated from two acidic lakes with distinct P availability. The strains were cultured in a mineral medium supplied with organic (beta-glycerol phosphate) or inorganic (orthophosphate) P at three concentrations. Both strains responded to experimental conditions in a similar way, suggesting that acid extracellular phosphatases were regulated irrespectively of the origin and history of the strains. We found an increase in cell-specific PA at low P concentration and the cultures grown with organic P produced significantly higher (ca. 10-fold) PA than those cultured with the same concentrations of inorganic P. The cell-specific PA measured in the cultures grown with the lowest organic P concentration roughly corresponded to those of the original *Coccomyxa* population from an acidic lake with impaired P availability. The ability of *Coccomyxa* strains to produce extracellular phosphatases, together with tolerance for both low pH and metals can be one of the factors enabling the dominance of the genus in extreme conditions of acidic lakes. The analysis of frequency distribution of the single-cell PA documented that simple visual counting of ‘active’ (labeled) and ‘non-active’ (non-labeled) cells can lead to biased conclusions regarding algal P status because the actual PA of the ‘active’ cells can vary from negligible to very high values. The FLEA assay using image cytometry offers a strong tool in plankton ecology for exploring P metabolism.

## Introduction

Phosphorus (P) has been proven to be a limiting resource in many aquatic ecosystems (Schindler, 2012; Schindler et al., 2016). Aquatic microorganisms, except for phagotropic protists, can only assimilate dissolved inorganic P, i.e., dissolved orthophosphate (P_i_) (Reynolds, 1997). Yet P_i_ also reacts with and adsorbs to various compounds or seston particles (e.g., clay) that may sediment and ultimately reduce the availability of P in the epilimnion and euphotic zone. Therefore, P_i_ is a subject of more or less severe competition in the planktonic microbial community, encompassing not only individual phytoplankton species (Sommer, 1981, 1985), but also bacterioplankton (Currie and Kalff, 1984; Cotner and Wetzel, 1992). On the other hand, plankton consumers may regenerate substantial amounts of P_i_ into the water column (e.g., Knoll et al., 2016). Such a consumer driven nutrient recycling often results in dissolved organic P (DOP) forms that are not readily available to microorganisms. The DOP compounds need to be cleaved by extracellular enzymes before they can be taken up by microbial cells (Cembella et al., 1984; Jansson et al., 1988; Cotner and Wetzel, 1991).

In the light of this, several artificial chromogenic or fluorogenic substrates have been used for regular measurements of the extracellular phosphatase activity (Jones, 1972; Healey and Hendzel, 1979; Hoppe, 1983), increased level of which in lake water was proposed to indicate P deficiency in lake phytoplankton (Healey and Hendzel, 1980). By adding an artificial DOP substrate to a water sample and to its cell-free filtrate, total and free (dissolved) phosphatase activities are measured, respectively. The free, or dissolved, activity represents the bulk activity of all free (dissolved) enzymes, both of microbial and metazoan origin (Boavida and Heath, 1984; Carr and Goulder, 1990). The particulate activity, calculated as the difference of total and free activity, represents the bulk activity of both microbial ectoenzymes and free enzymes adsorbed to particles (Wetzel, 1991; Nagata and Kirchman, 1992). It is sometimes possible to estimate proportions of ‘bacterial’ and ‘algal’ ectoenzymes by using a more detailed size fractionation of water samples (Vrba et al., 1993; Nedoma et al., 2006); however, none of the widely-used substrates allows for enzyme localization or detection of phosphatase producers, which is the serious methodological drawback of the bulk phosphatase assay.

A new generation of fluorogenic substrates, such as ELF^®^ 97 phosphate (ELFP) based on 2-(2′-phosphoryloxyphenyl)-4-(3H)-quinazolinone (Huang et al., 1992), can overcome most disadvantages. Insoluble precipitates of the hydrolysis product (ELF^®^ 97 alcohol, ELFA) at the sites of hydrolysis (under certain conditions – see below) allow for direct visualization of the active enzymes in organisms (cells) by epifluorescence microscopy. An early application of the ELF^®^ 97 Endogenous Phosphatase Detection Kit successfully visualized phosphatase-positive cells in both algal cultures and natural phytoplankton, i.e., directly tagged the P-limited algal species (González-Gil et al., 1998; Dyhrman and Palenik, 1999; Rengefors et al., 2001), although further studies revealed some uncertainties and/or potential misinterpretations (e.g., Rengefors et al., 2003; Dignum et al., 2004; Ou et al., 2010). The principle shortcoming of the ELF^®^ 97 method as it is applied in recent studies, including the original paper by González-Gil et al. (1998), is that only the occurrence of ELFA-labeling, i.e., merely qualitative estimates of phosphatase-positive algal cells and/or species, presence/absence of tagged phytoplankton, etc., could be reported (Štrojsová et al., 2003; Cao et al., 2005; Rychtecký et al., 2015; Ren et al., 2017). The only quantification of phosphatase activity accessible within the limits of this original method is to score a percentage of ELFA-labeled cells (e.g., Rengefors et al., 2003; Dyhrman and Ruttenberg, 2006; Litchman and Nguyen, 2008; Young et al., 2010).

This problem has been largely solved by using ELF^®^ 97 phosphate (ELFP) according to a modified protocol (Nedoma et al., 2003b; Štrojsová et al., 2003) derived from a common fluorescence assay (e.g., Hoppe, 1983) for extracellular activity in plankton and further standardized by buffering the samples (Štrojsová and Vrba, 2006). Inhibition experiments suggested that both substrates (i.e., ELFP and 4-methylumbelliferyl phosphate) were hydrolyzed by the same extracellular phosphatases (Štrojsová et al., 2003). This protocol, referred to as fluorescence-labeled enzyme activity (FLEA) assay, allows not only for distinguishing between enzymatically active and inactive specimens in a sample, but, most essentially, for the quantification of the ELFA fluorescence at single cell or species level using image cytometry (Nedoma et al., 2003b; Nedoma and Vrba, 2006; Novotná et al., 2010). This cell-specific fluorescence intensity can be further converted to a specific rate of enzymatic ELFP hydrolysis by the particular producers.

Since decades ago, realistic interpretation and sometimes contradictory results of various phosphatase assays remains a subject of discussions in plankton ecology (e.g., Berman et al., 1990; Nedoma et al., 2003a). There is no doubt, at present, that the bulk extracellular phosphatase activity must not be interpreted as exclusively algal activity (e.g., Hoppe, 2003; Cao et al., 2005; Nedoma et al., 2006). The paradigm of phosphatase expression only under P deficiency is overly simplistic as algae may constitutively express some phosphatase activity, but also may not efficiently regulate it in response to P availability (Young et al., 2010). There is also increasing awareness that all phytoplankton species do not react uniformly to P depletion (e.g., Litchman and Nguyen, 2008) and many species indeed do not produce extracellular phosphatases at all under such circumstances, while other species can exhibit constitutive activity (e.g., Rengefors et al., 2001, 2003; Štrojsová et al., 2003, 2005, 2008; Rychtecký et al., 2015). For instance, ELFA-labeled, i.e., phosphatase-positive phytoplankton species were reported from eutrophic lakes under high concentrations of soluble reactive P (SRP) (e.g., Cao et al., 2005, 2009). Most data, however, have been obtained from field studies. Laboratory experiments focused on the influence of P form and concentration on cell-specific phosphatase activity under controlled conditions are scarce and performed entirely in batch cultures (Huang et al., 2000; Young et al., 2010; Ren et al., 2017).

In this study, we examined two closely related algal species isolated from two acidic lakes differing in their P concentrations. Each algal population had been exposed to distinct environmental conditions for decades. We tested the ability of both species to grow on inorganic or organic P in a semi-continuous system, and the response of single algal cells to various degree of P depletion. Cell-specific acid phosphatase activity was measured using the FLEA assay according to the protocol, which enabled us to quantify more accurately its variability in individual experimental treatments. The main objectives of this study were to determine (i) if expression of algal acid phosphatases is under environmental control, (ii) if the manner of the control differs in the isolates originating from environments contrasting in P availability, and (iii) if acid phosphatase activity reflects actual needs of algal cells given by their growth rate and source of P.

## Materials and Methods

### Algal Cultures

We isolated two unialgal cultures of *Coccomyxa* strains (Trebouxiophyceae, Chlorophyta) by serial dilution from the plankton of two acidic lakes of distinct trophic status in Czechia. Lake Plešné (48°46′35″ N, 13°51′55″ E; 1087 m a.s.l.) is of glacial origin and it was strongly acidified due to atmospheric sulfur and nitrogen deposition that peaked in the 1980s (Vrba et al., 2003). In this acidic (pH = 4.8–5.5) mesotrophic lake, P availability remains largely impaired by reactive aluminum (Vrba et al., 2006), with mean epilimnetic SRP concentrations as low as ∼40 nmol L^-1^ (Novotná et al., 2010). Its P-limited phytoplankton are dominated by coccoid green algae (formerly misidentified as *Monoraphidium dybowskii*; cf. Štrojsová and Vrba, 2006, 2009) that was recently described as a new species, *Coccomyxa silvae-gabretae* (Barcytė and Nedbalová, 2017). Eutrophic Lake Hromnice (49°51′03″ N, 13°26′39″ E; 330 m a.s.l.) is a former pyritic shale mine. Its lake water is characterized by extremely low pH (2.3-2.9), high concentrations of P (1-52 μmol L^-1^ SRP) and several metals (Al, Fe, Mn, Ni, Cu, Co, and Pb) (Hrdinka et al., 2013). A common phytoplankton species in Lake Hromnice is *Coccomyxa elongata* (Barcytė and Nedbalová, 2017). We maintained non-axenic cultures of the two strains in an acidified BBM medium (Bischoff and Bold, 1963), with the pH adjusted to 4, at room temperature and daylight.

For all phosphatase experiments, we cultivated both *Coccomyxa* strains in semi-continuous, turbidostatic systems, in the acidified BBM medium supplied with distinct P sources at three concentrations (see below), at room temperature and permanent light provided by fluorescent tubes (photosynthetically active radiation ∼40 μmol s^-1^ m^-2^). We used 0.5-L conical vessels (separatory funnels with stopcock for easy sampling), filled with 200 ml of medium and inoculated with 0.5 ml of stock culture at the beginning of each experiment. The medium as well as cultivation vessels were sterilized. Continuous aeration by sterile air bubbling into the bottom of each vessel ensured both CO_2_ saturation and mixing of algal suspension. To provide merely inorganic (hereafter referred as I) or organic (hereafter referred as O) P sources, we supplied the BBM medium with P_i_ or β-glycerol phosphate (β-GP) as the single source of P, respectively. For either source, we used one P-replete (variants I1 and O1) and two P-depleted (variants I2-I3 or O2-O3) media with the original concentrations adjusted to 858, 16 and 10 μmol L^-1^ of P.

We ran all experimental variants in triplicates for 3 weeks. We regularly screened all variants for chlorophyll *a* concentration using a fluorometer (TD-700 Laboratory Flurometer, Turner Designs, San Jose, CA, United States) and diluted the cultures by the corresponding fresh medium at regular intervals, i.e., three times during the cultivation, to maintain chlorophyll *a* concentrations close to ∼10 μg L^-1^ in P-depleted (I2/O2 and I3/O3) and to 50 μg L^-1^ in P-replete (I1/O1) variants. In addition, in the middle and at the end of each experiment, we checked all variants for residual P concentrations in cultures – SRP was determined by the molybdate method after filtering the samples through glass fiber filters (0.7 μm, Macherey-Nagel, Düren, Germany). After the 3-week cultivation, we sampled all replicates to estimate cell-specific phosphatase activity of individual *Coccomyxa* populations in each experimental variant.

For each cultivation, we further calculated a specific growth rate (μ, day^-1^) of the individual *Coccomyxa* population for the period between the second and third dilutions according to the equation:

$$\begin{array}{c} \mu =\frac{lnN_{f}-lnN_{i}}{t_{f}-t_{i}} \end{array}$$

where N is the final (f) or initial (i) cell density at time (t). A conversion curve was used to calculate N from chlorophyll fluorescence values.

### Cell-Specific Phosphatase Activity (FLEA Assay)

After the 3-week cultivations, we employed the protocol for FLEA assay (Nedoma et al., 2003b) to estimate extracellular cell-specific phosphatase activity of the *Coccomyxa* strains grown on different P sources. We incubated 5-ml samples with fluorogenic substrate ELFP (Molecular Probes; Invitrogen, Eugene, OR, United States). The incubation started by the addition of ELFP solution (final concentration of 20 μmol L^-1^) and lasted 3 h at room temperature and daylight. Then, each incubation was terminated by filtering 1-ml subsamples over mild vacuum (<20 kPa) through polycarbonate membrane filters (pore size 2 μm; Osmonics, Minnetonka, MN, United States). The filter with retained algae was placed on a microscopic slide, embedded with immersion oil, covered with a coverslip, and preserved in a freezer at -20°C until the image cytometry analysis (cf. Nedoma et al., 2003b).

### Image Cytometry

**FIGURE 1:**
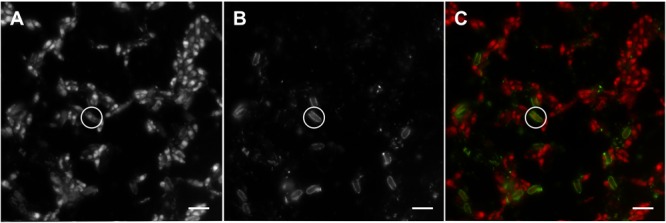
A set of epifluorescence microphotographs of the *Coccomyxa elongata* culture with induced extracellular phosphatase activity grown in P-depleted organic medium (O3). Same image areas show: **(A)** the image captured with chlorophyll-autofluorescence-specific filter cube (excitation/emission: 510–550 nm/>590 nm) visualizing single algal cells; **(B)** the image captured with ELFA-fluorescence-specific filter cube (excitation/emission: 360–370 nm/520–540 nm) visualizing fluorescence-labeled enzyme activity (FLEA), i.e., the ELFA precipitates on surfaces of phosphatase-positive cells; and **(C)** the merged images A and B in artificial colors – red and green stand for chlorophyll autofluorescence and ELFA fluorescence, respectively. Circles indicate the same phosphatase-active cell; scale bars represent 10 μm.

The image analysis system used for ELFA fluorescence quantification included the fluorescence microscope Nikon Eclipse 90i (Nikon, Tokyo, Japan; Nikon Plan Fluor 60×), monochromatic digital camera (Andor Clara, Andor Technology, Ltd., Belfast, United Kingdom), and the software NIS-Elements 4.12 (Laboratory Imaging, s.r.o., Prague, Czechia). From every slide, 30 image files corresponding to 30 randomly selected microscope fields were made. Each image file contained two types of images from two channels (**Figure 1**) – one was captured with ELFA-fluorescence-specific filter cube (excitation/emission: 360–370 nm/520–540 nm) and served for the measurement of cell-associated ELFA fluorescence; the second image was captured with chlorophyll-autofluorescence-specific filter cube (excitation/emission: 510–550 nm/>590 nm) and served for cell localization and sizing (see below). In NIS-Elements software, 3-6 randomly chosen cells (90-180 from one slide) were demarcated (segmented) manually on the chlorophyll-fluorescence image. The system then measured cell dimensions and the mean gray level of the cell and of the ELFA image background. Cell-associated ELFA fluorescence [F_ELFA_, in relative fluorescence units (FUs) cell^-1^h^-1^] was then calculated using the following equation (Nedoma et al., 2003b):

$$\begin{array}{c} F_{\mathrm{ELFA}}=\frac{\mathrm{Area}\times (\mathrm{MGrey}-\mathrm{BgMGrey})}{T_{\exp}}\times F_{\mathrm{cal}} \end{array}$$

where Area (μm^2^) is projected area of the cell, MGrey (dimensionless) is mean gray of the cell, BgMGrey (dimensionless) is mean gray of the background, F_cal_ (dimensionless) is fluorescence calibration factor, and T_exp_ (ms) is exposure time. For rough estimation of the cell-specific phosphatase activity (in the units of fmol cell^-1^h^-1^), we used the conversion factor of 0.1 fmol FU^-1^, based on experiments with Plešné Lake natural plankton (for details see Nedoma et al., 2003b).

Mean cell volumes of individual *Coccomyxa* populations in each replicate were calculated using the measured cell dimensions, i.e., cell length and area from the chlorophyll-fluorescence images, by approximation of cell shape to an ellipsoid.

### Statistical Analyses

A three-way ANOVA with a *post hoc* Tukey HSD test of differences among experimental variants were performed to test the effects of P sources, P concentrations, species of *Coccomyxa*, and their interactions on algal growth rate, mean cell size, and cell-specific phosphatase activity. All data were transformed by log (x+1) to meet the assumptions of ANOVA. All analyses were performed using Statistica 13.2 (Dell Inc., 2016).

## Results

The two strains of *Coccomyxa* species revealed very similar results and generally responded in a consistent way to all experimental treatments. No significant differences in either of the treatments were detected between both species (**Tables 1–3**). Final residual concentrations averaged at around 600 μmol L^-1^ of SRP in P-replete cultures grown on inorganic medium (P_i_, I1), whereas they leveled at ∼30 μmol L^-1^ of SRP in those grown on organic medium (β-GP, O1). Yet, in all P-depleted variants, these concentrations were very similar, on average 2–6 μmol L^-1^ of SRP. In the organic media, β-GP was obviously transformed into SRP in all treatments.

**Table 1:** Results of three-way ANOVA testing the effects of species (*C. elongata* vs. *C. silvae-gabretae*), media (inorganic vs. organic), and three P concentrations on growth rate.

Factor	df	*F*	*P*
Species (S)	1	0.02	0.9
Medium (M)	**1**	**4.69**	**0.04**
P concentration (P)	**2**	**44.0**	**<0.001**
S × M	1	0.88	0.36
S × P	2	0.88	0.43
M × P	2	0.59	0.56
S × M × P	2	1.19	0.32

*Significant differences (P < 0.05) are given in bold; ×, interaction of factors*.

**Table 2:** Results of three-way ANOVA testing the effects of species (*C. elongata* vs. *C. silvae-gabretae*), media (inorganic vs. organic), and three P concentrations on cell volume.

Factor	df	*F*	*P*
Species (S)	1	0.61	0.44
Medium (M)	1	0.14	0.71
P concentration (P)	**2**	**11.3**	**<0.001**
S × M	1	0.61	0.44
S × P	2	0.55	0.58
M × P	2	0.26	0.77
S × M × P	2	0.07	0.93

*Significant differences (P < 0.05) are given in bold; ×, interaction of factors*.

**Table 3:** Results of three-way ANOVA testing the effects of species (*C. elongata* vs. *C. silvae-gabretae*), media (inorganic vs. organic), and three P concentrations on cell-specific phosphatase activity.

Factor	df	*F*	*P*
Species (S)	1	0.02	0.88
Medium (M)	**1**	**36.3**	**<0.001**
P concentration (P)	**2**	**32.8**	**<0.001**
S × M	1	0.64	0.43
S × P	2	0.03	0.97
M × P	**2**	**9.66**	**<0.001**
S × M × P	2	0.42	0.66

*Significant differences (P < 0.05) are given in bold; ×, interaction of factors*.

**FIGURE 2:**
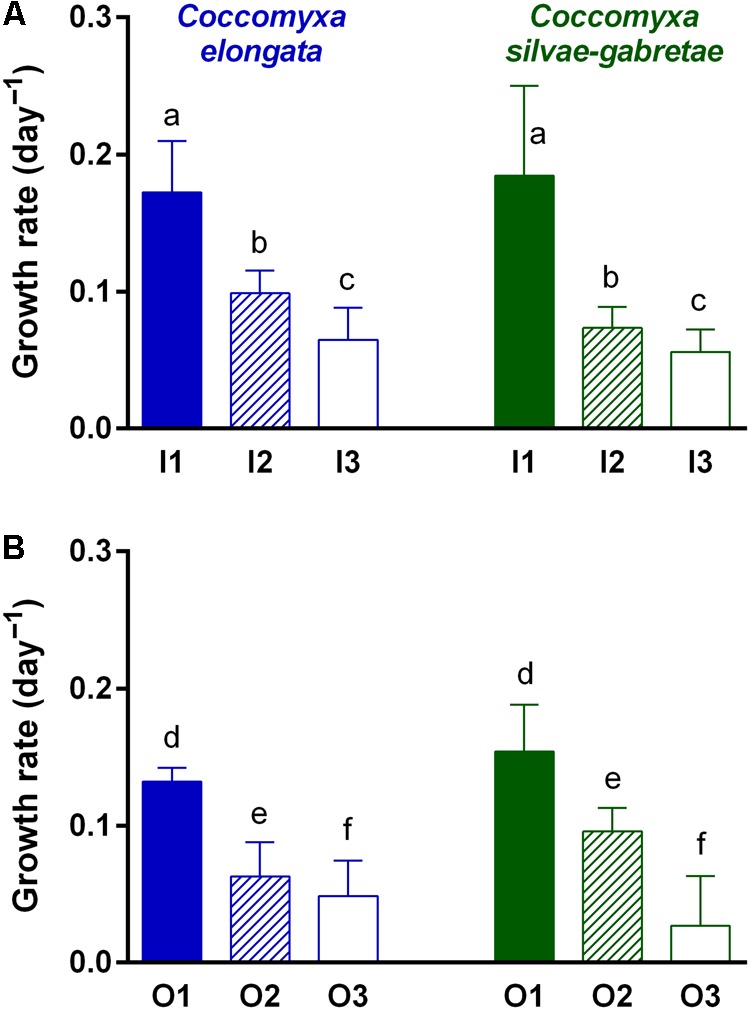
Comparison of growth rates of the *Coccomyxa* cultures in P-replete (1) and P-depleted (2 and 3) media with either inorganic (I; top: **A**) or organic (O; bottom: **B**) P source. Columns are means, bars represent SDs; note that scales are the same. Differences among treatments were tested using three-way ANOVA with a *post hoc* Tukey HSD test; lower case letters above the columns (a–f) indicate significant differences among treatments (see **Table 1** for summary statistics).

**FIGURE 3:**
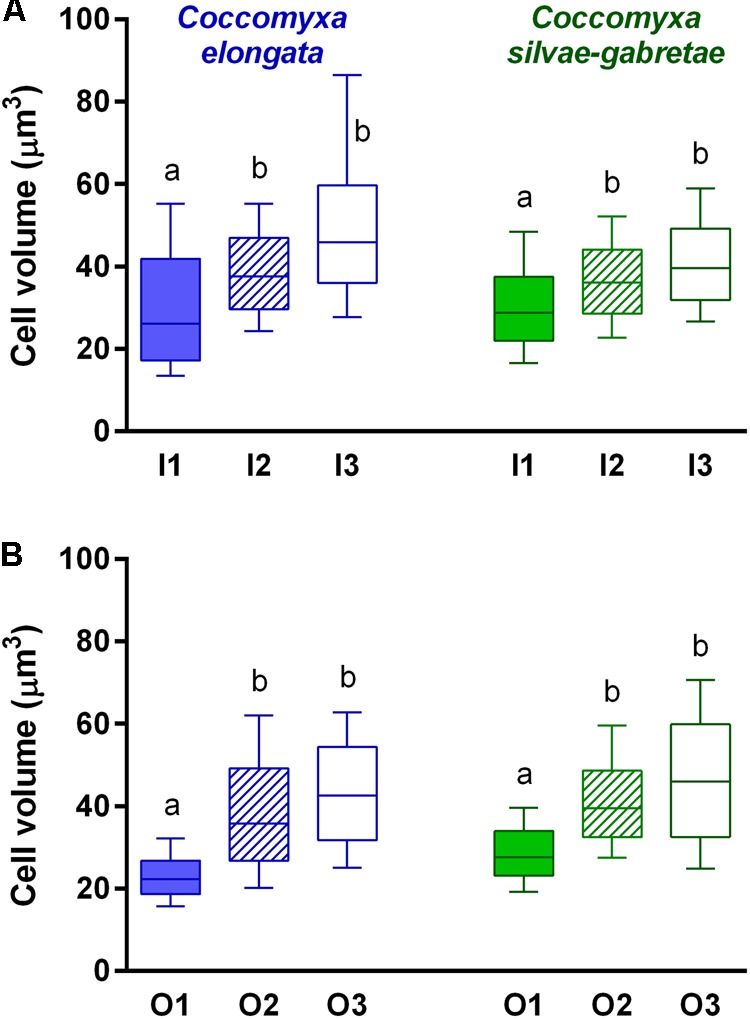
Comparison of mean cell volume of the *Coccomyxa* cultures in P-replete (1) and P-depleted (2 and 3) media with either inorganic (I; top: **A**) or organic (O; bottom: **B**) P source. Box and whisker plots show medians (bar), 25 and 75% quartiles (box), and 10–90% percentiles (whiskers); note same scales. Differences among treatments were tested using three-way ANOVA with a *post hoc* Tukey HSD test; lower case letters above the columns (a, b) indicate significant differences among treatments (see **Table 2** for summary statistics).

We found the highest growth rates (0.17–0.18 day^-1^) in P-replete P_i_ medium (variants I1), whereas they were significantly lower (0.06–0.10 day^-1^) in both P-depleted variants (I2 and I3; **Figure 2**). Moreover, both the species reached significantly lower growth rates in media with β-GP (0.13–0.15 and 0.03–0.10 day^-1^, respectively) compared to their P_i_ counterparts (**Table 1**), while keeping the same descending trends from P-replete to depleted variants (O1–O3; **Figure 2B**). Notwithstanding the P source and species, all P-depleted cultures revealed significantly larger mean cell volumes (38–52 μm^3^) than those that were P-replete (23–31 μm^3^; **Figure 3** and **Table 2**).

**FIGURE 4:**
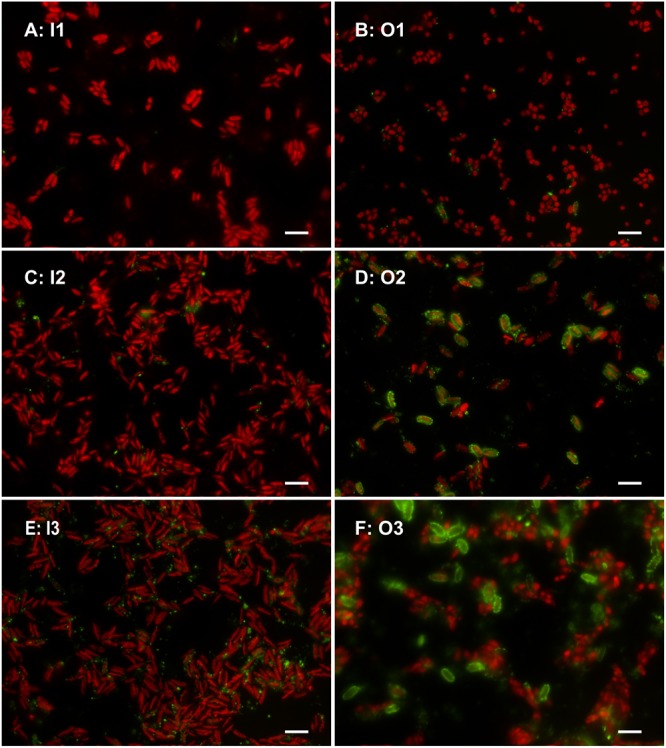
Microphotographs of the *C. elongata* cultures grown in all experimental treatments (see variants’ codes), i.e., in P-replete (top: **A,B**) and P-depleted (middle and bottom: **C–F**) media with inorganic (left) or organic (right) P sources. Combined images in artificial colors (for details, see **Figure 1**); scale bars represent 10 μm.

**FIGURE 5:**
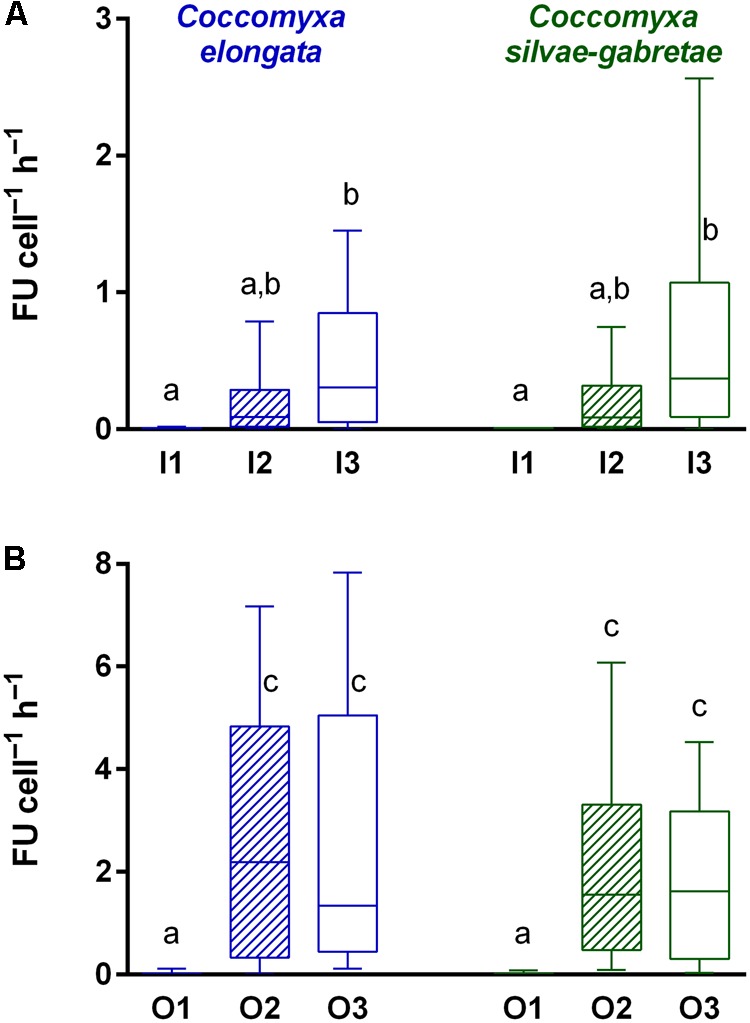
Comparison of cell-specific phosphatase activity [in relative fluorescence units (FUs)] in the *Coccomyxa* cultures in P-replete (1) and P-depleted (2 and 3) media with either inorganic (I; top: **A**) or organic (O; bottom: **B**) P source. Box and whisker plots show medians (bar), 25 and 75% quartiles (box), and 10 and 90% percentiles (whiskers); note the different scales of y axes. Differences among treatments were tested using three-way ANOVA with a *post hoc* Tukey HSD test; lower case letters above the columns (a–c) indicate significant differences among treatments (see **Table 3** for summary statistics).

In both P-replete media, we detected negligible ELFA labeling (**Figures 4A,B**) in both *Cocomyxa* cultures. We therefore estimated close to zero cell-specific phosphatase activity (relative FU cell^-1^h^-1^) in every replicate of the I1 and O1 variants (**Figure 5**). On the contrary, we detected substantial phosphatase activity in all P-depleted cultures. While its increase, compared to the corresponding P-replete variant, was lower in P-depleted P_i_ media and significant only in the I3 variant, all P-depleted cultures grown with β-GP exhibited bright fluorescence (**Figures 4C–F**). Moreover, the cell-specific phosphatase activities in O2 and O3 treatments exceeded those in I2 by one order of magnitude (**Figure 5**). Three-way ANOVA confirmed highly significant effects of both P source and P concentration, as well as their interaction (see, respectively, factors M and P in **Table 3**). This interaction reflected different responses to the P source concertation in the P_i_ and β-GP cultures (cf. **Figures 5A,B**).

**FIGURE 6:**
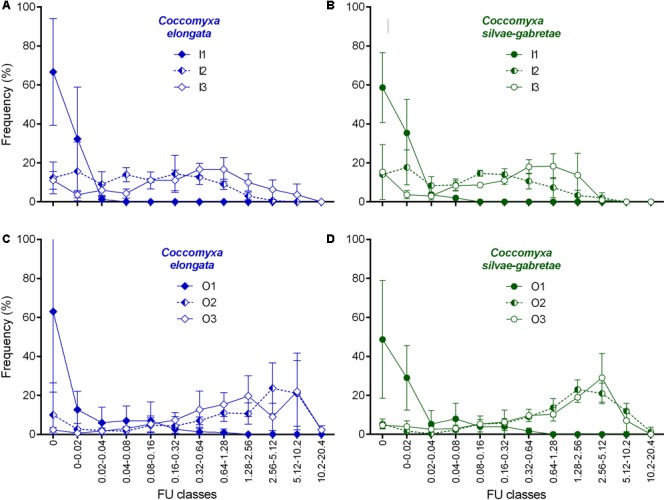
Frequency distribution of cell-specific phosphatase activity (in relative FU cell^-1^h^-1^) in the *Coccomyxa* cultures in P-replete (1) and P-depleted (2 and 3) media with either inorganic (I; top: **A,B**) or organic (O; bottom**: C,D**) P source. Except for zero classes (no activity), all FU classes are expressed in a geometric progression to cover broad range of ELFA fluorescence. All symbols are means of triplicates, bars represent SDs; note same scales; 100% is the sum of means in a variant. The total cell number (in a variant) measured for *C. elongata*: 300 (I1), 360 (I2), 420 (I3), 357 (O1), 360 (O2), 360 (O3), and *C. silvae-gabretae*: 300 (I1), 360 (I2), 355 (I3), 355 (O1), 359 (O2), 360 (O3).

We further analyzed the frequency distributions of the cell-specific phosphatase activities measured in all replicates of each treatment. In general, we did not find any remarkable difference in the distribution patterns among the two *Coccomyxa* species tested (**Figure 6**). In the P-replete cultures, most of the algal cells (nearly 100% in I1 and almost 80% in O1) exhibited negligible activity (<0.02 FU cell^-1^h^-1^). Unlike in I1 variants, up to ∼20% of algae in the O1 cultures exhibited low activity (<0.64 FU cell^-1^h^-1^). On the contrary, we observed low percentage of such weakly ELFA-labeled and/or inactive cells with very similar distribution patterns in all P-depleted β-GP cultures (cf. O2 and O3 in **Figures 6C,D**). The P-depleted P_i_ and β-GP cultures, however, showed very different distribution patterns in two aspects: (i) the maximum in the histogram of single-cell phosphatase activities was notably shifted toward higher activities in β-GP compared to P_i_ cultures (peaking around 0.6 and 2.5 FU cell^-1^h^-1^, respectively), and (ii) in P_i_ cultures, the moderate P-depletion (I2) resulted in a flat and uniform frequency distribution limited to the region of low activities (<1.26 FU cell^-1^h^-1^), whereas the high P-depletion (I3) induced clear maximum between 0.32 and 1.26 FU cell^-1^h^-1^ (**Figures 6A,B**).

**FIGURE 7:**
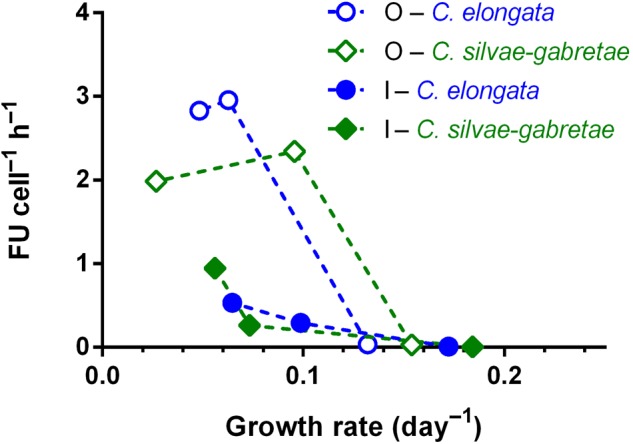
Relationship of cell-specific phosphatase activity (in relative FUs) to the growth rates of *Coccomyxa* cultures grown with inorganic (I) or organic (O) P source. Symbols are means of triplicates.

At comparable growth rates, the cell-specific phosphatase activities were roughly 5–10 times higher in the variants with β-GP compared to P_i_ as phosphorus source. The relationship between growth rate and phosphatase activity was similar in both *Coccomyxa* species examined (**Figure 7**).

## Discussion

Our results clearly suggest that both tested *Coccomyxa* species, although their original populations had lived in acidic lakes with the contrasting P availability for decades, possessed the same ability to produce acid extracellular phosphatases. We can speculate that the absence of a genomic adaptation to high P concentrations in *C. elongata* indicates that the production of acid phosphatases represent an evolutionarily conservative trait of vital importance for the acidotolerant algae. These phosphatases were inducible ectoenzymes (cf. Chróst, 1991), exclusively produced in all P-depleted cultures, while their production in the P-replete variants (I1 or O1) was negligible. Some early studies considered alkaline phosphatases as inducible and acid phosphatases as constitutive (Cembella et al., 1984; Jansson et al., 1988). In contrast, individual phytoplankton species exhibited, depending on circumstances, zero to extreme acid phosphatase activity per cell in chronically P limited acidic lakes, indicating that these ectoenzymes were inducible too (Štrojsová and Vrba, 2009; Novotná et al., 2010). Our results in this study suggested that acid phosphatases in *Coccomyxa* species were regulated in the same manner as it is known for alkaline phosphatases (e.g., Jansson et al., 1988).

Surprisingly, the relatively well-growing O1 cultures, grown entirely with organic P source, produced very little phosphatases. Most likely, some non-enzymatic hydrolysis of β-GP could liberate enough P_i_ for algal growth, as suggested by the residual SRP concentrations (∼30 μmol L^-1^) observed in this P-replete medium (O1). The high β-GP concentration could also saturate the enzymes to such a degree that the ELFP substrate was outcompeted during the assay (likewise glucose-6-phosphate and 4-methylumbelliferyl phosphate inhibited the ELFP hydrolysis; cf. Figure 1 in Štrojsová et al., 2003). Consequently, just <20% of algal cells showed weak ELFA labeling (**Figure 6**). Moreover, we could not exclude some β-GP hydrolysis by bacterial extracellular enzymes (cf. Siuda and Chróst, 2001) as the algal cultures were not axenic. Such an enzymatic activity, however, would not interfere with the FLEA assay, which specifically quantifies relative fluorescence of individual algae (**Figure 1**). Furthermore, hardly any bacterial or free activity would be retained on the filter used (2-μm pore size); indeed very few such ELFA precipitates were observed by epifluorescence microscopy (**Figure 4B**).

Cell-specific phosphatase activities were almost an order of magnitude higher with β-GP (O2 and O3) compared to those with P_i_ (I2 and I3) (cf. the different scales in **Figures 5A,B**) and the growth rates with P_i_ were slightly but significantly higher than those with β-GP (**Figure 2** and **Table 1**). Similar responses were recently reported also by Ren et al. (2017), who cultured algal (*Chlorella pyrenoidosa* and *Pseudokirchneriella subcapitata*) or cyanobacterial (*Microcystis aeruginosa*) species with various P sources in axenic batch cultures. Similarly to our study, both green algae (*C. pyrenoidosa* and *P. subcapitata*) and cyanobacteria grew faster with P_i_ than with β-GP or glucose-6-phosphate (Ren et al., 2017). At the same P concentrations, P_i_ provided apparently better support for growth than organic P sources (**Figure 7**). Hence, the production of phosphatases might represent additional investment of energy (Novotná et al., 2010) and/or the phosphatases were not able to liberate enough P_i_ for growth. The substantially higher phosphatase activity in the cultures grown with organic P could reflect stronger P deficiency in these cultures compared to those grown with P_i_. Besides, not only the lack of P_i_ but also the presence of organic P could contribute to phosphatase upregulation. In other words, both *Coccomyxa* species maintained approximately twofold higher phosphatase activity to perform the growth rates lower or equal to 0.1 day^-1^ as shown in **Figure 7**.

Our results suggested fully inducible nature of acid phosphatases in the studied algae, because ELFA labeling was negligible in either P_i_ or β-GP excess. On the contrary, Young et al. (2010) observed certain P-insensitive component of alkaline phosphatase activity in the benthic *Cladophora*-epiphyte assemblage from Lake Michigan, cultured with P_i_ and α-glycerol phosphate supply, as well. Their conclusions, however, were based on an experimental study on the benthic assemblage, i.e., neither planktonic nor unialgal populations (Young et al., 2010). Moreover, their conclusions were based only on qualitative evidence (i.e., presence of ELFA-labeling) and not on quantification of the cell-specific phosphatase activity as was the case in this and other studies on *C. silvae-gabretae* (Štrojsová and Vrba, 2006, 2009; Novotná et al., 2010).

In our study, all P-depleted *Coccomyxa* cultures had significantly higher mean cell volumes compared to those that were P-replete (**Figure 3**). Such larger cells of P-limited algae were reported in batch cultures of *Scenedesmus quadricauda* and *Asterionella formosa* (Litchman and Nguyen, 2008), as well as in continuous cultures of *Cryptomonas phaseolus* (Mindl et al., 2005). Cells in the P-limited cultures likely divided less often due to the lack of P and, at the same time, enlarged their volume via storing photosynthates. The larger cells indeed should result in a relatively low P content, hence, in other words, in the low cellular P quota and, consequently, in high phosphatase expression (Litchman and Nguyen, 2008).

Unlike in the short-term experiments with gradually exhausting sources in the batch cultures (e.g., Ou et al., 2010; Ren et al., 2017), in the present study, we employed semi-continuous cultures, better adjusted to keep the (limiting) P concentration tested. This setup allowed us to maintain the algal cultures at a more stable P deficiency or P sufficiency for longer periods of time, to maintain more close-to-natural conditions of both original *Coccomyxa* populations (Barcytė and Nedbalová, 2017), and to allow for their cross-comparison. While *C. elongata* dominated in the phytoplankton of the eutrophic Lake Hromnice (Hrdinka et al., 2013), *C. silvae-gabretae* (previously misdetermined as *Monoraphidium dybowskii*) prevailed in the phytoplankton biomass of the mesotrophic Lake Plešné, with enormous bulk activity of extracellular phosphatases due to impaired P availability (Vrba et al., 2006).

Our results on *C. silvae-gabretae* cultures in this study are consistent with the observed *in situ* response of this species to increasing P availability. The *in situ* phosphatase activity of *C. silvae-gabretae* gradually decreased with the progressing lake recovery from acid stress during past decades (Novotná et al., 2010). The first application of FLEA assay in Lake Plešné in 2001 showed remarkable ELFA-labeling of all bacterioplankton, as well as of most phytoplankton species, including the entire population of *C. silvae-gabretae* (Nedoma et al., 2003b). The cell-specific phosphatase activity of this species varied within four orders of magnitude (0.5–500 fmol cell^-1^h^-1^) in 2003 (Štrojsová et al., 2005), but averaged within a closer range (0.4–12 fmol cell^-1^h^-1^) in 2005 (Štrojsová and Vrba, 2009), whereas hardly any production of extracellular phosphatases, i.e., weak ELFA-labeling of *C. silvae-gabretae* cells, was detected in 2007 (Novotná et al., 2010). Using a conversion factor of 0.1 fmol FU^-1^ for our experimental results (in relative FU cell^-1^h^-1^), medians of phosphatase activity in the P-depleted variants I3 and O3 (**Figure 5**) were 0.04 and 0.16 fmol cell^-1^h^-1^, respectively, and the most frequent classes in both O2 and O3 variants (**Figure 6D**) fell between 1.3 and 5.1 fmol cell^-1^h^-1^ for the strain of *C. silvae-gabretae*. These estimates of cell-specific phosphatase activity corresponded well to those determined for growing native populations of *C. silvae-gabretae* in Lake Plešné during the period of severe P limitation (Nedoma et al., 2003b; Štrojsová et al., 2005; Štrojsová and Vrba, 2009). The same estimates were calculated for the strain of *C. elongata* as well, although its native population in Lake Hromnice likely never experienced serious P depletion (Hrdinka et al., 2013).

The residual SRP concentrations in all P-depleted experimental variants (2–6 μmol L^-1^) were at least by one order of magnitude higher than the threshold indicating P limitation in freshwaters (∼0.15 μmol L^-1^ of SRP; Nedoma et al., 1993; Vrba et al., 1993), whereas the epilimnetic SRP concentrations in Lake Plešné averaged seasonally as low as 0.04 μmol L^-1^ (Novotná et al., 2010). Yet in this lake, Štrojsová and Vrba (2009) documented remarkable diurnal variations in cell-specific phosphatase activity within the native population of *C. silvae-gabretae* in 2005, while Novotná et al. (2010) could not detect any measurable activity in this species at all in 2007. Both field studies suggested that single cells in the phytoplankton populations may differ remarkably in their individual cell-specific phosphatase activities due to the asynchronous character of the populations. Single algal cells likely reflected their internal needs in P, i.e., individual cellular P quota, as also suggested by Litchman and Nguyen (2008) or Ren et al. (2017). Štrojsová and Vrba (2009) proposed that distinct sub-populations (such as epilimnetic and metalimnetic) of *C. silvae-gabretae* with different life history characteristics could occur in the lake phytoplankton (e.g., due to strong mixing). Novotná et al. (2010) explained the absence of ELFA-labeling in the *C. silvae-gabretae* population by a pronounced P regeneration by grazing of abundant zooplankton, which re-colonized the lake between 2005 and 2007.

In our opinion, there is a common, but serious, methodological limitation in many recent studies employing ELFP (Rengefors et al., 2003; Dyhrman and Ruttenberg, 2006; Litchman and Nguyen, 2008; Young et al., 2010). Our analysis of frequency distribution of cell-associated ELFA fluorescences measured in this study (**Figure 6**) clearly illustrates the weakness of many studies employing ELFP that were based just on the scoring of ELFA-labeled cells (Rengefors et al., 2003; Dyhrman and Ruttenberg, 2006; Litchman and Nguyen, 2008; Young et al., 2010). For instance, our analysis showed that some ELFA-labeled cells could be found even in the P-replete culture (I1) grown on P_i_ (about some 40% of cells were ‘positive,’ i.e., with non-zero fluorescence; **Figure 6**), which could lead to a false conclusion that the population was P-deficient. Yet their actual phosphatase activity was negligible (in the range of 0–0.08 FU cell^-1^h^-1^; cf. **Figures 5A** and **6A** or **6B**). At present, the Fluorescently-Labeled Enzyme Activity assay (the true FLEA assay – Štrojsová and Vrba, 2006) is the only method available for adequate quantification of phosphatase activity at the level of individual cells or populations. The data on the percentages of ELFA-labeled cells should be interpreted with caution and considered, at the best, as semi-quantitative estimates of phosphatase activity in the assemblages (cf. Young et al., 2010).

Our experimental study suggested that the conclusions of our former field studies were plausible and confirmed the FLEA assay as a strong tool in phytoplankton ecology to explore P metabolism. To obtain reliable results, however, one should keep the following methodological recommendations: (i) Using ELF^®^ 97 phosphate as the substrate for algal extracellular phosphatases, because an application of the ELF^®^ 97 Endogenous Phosphatase Detection Kit likely may cause permeability of cell membranes and result in tagging of intracellular enzymes in some species (cf. Dyhrman and Palenik, 1999). (ii) Buffering phytoplankton samples, if pH *in situ* exceeds 8, to ensure the precipitation of ELFA molecules (Štrojsová and Vrba, 2006). (iii) Terminating sample incubation by gentle filtration (without application of phosphate buffered saline); only preserving samples with HgCl_2_ before filtration is recommended for fragile flagellates (Nedoma et al., 2003b; Štrojsová et al., 2003). (iv) The ELFA-fluorescence-specific filter cube (see section “Materials and Methods”) should be used for acquiring images; another chlorophyll-autofluorescence-specific filter cube is recommended to localize algal cells (**Figure 1**). A monochromatic rather than color camera is optimal for convenient image cytometry.

## Conclusion

The application of the FLEA assay in this experimental study confirmed the existence of environmental control of extracellular phosphatase expression in some acidotolerant algae and provided an insight into the impact of different P forms and concentrations on phosphatase activity in phytoplankton. This study represents the first evidence of inducible nature of acid phosphatases in algae. Our results further stress the importance of careful application of the FLEA method to gain reliable quantification of phosphatase activity at the single cell level.

## Author Contributions

JV, MM, and LN designed the experiment. MM and JN performed the image analysis. LN and MŠ performed the statistical analyses. All authors contributed to the manuscript.

## Conflict of Interest Statement

The authors declare that the research was conducted in the absence of any commercial or financial relationships that could be construed as a potential conflict of interest.

## References

[B1] Barcytė D. Nedbalová L. (2017). *Coccomyxa*: a dominant planktic alga in two acid lakes of different origin. *Extremophiles* 21 245–257. 10.1007/s00792-016-0899-6 27942983

[B2] Berman T. Wynne D. Kaplan B. (1990). Phosphatases revisited: analysis of particle-associated activities in aquatic systems. *Hydrobiologia* 207 287–294. 10.1007/BF00041467

[B3] Bischoff H. W. Bold H. C. (1963). *Phycological Studies. IV. Some Soil Algae from Enchanted Rock and Related Algal Species.* Austin, TX: University of Texas Publications.

[B4] Boavida M. J. Heath R. T. (1984). Are the phosphatases released by *Daphnia magna* components of its food? *Limnol. Oceanogr.* 29 641–645. 10.4319/lo.1984.29.3.0641

[B5] Cao X. Song C. Zhou Y. Štrojsová A. Znachor P. Zapomělová E. (2009). Extracellular phosphatases produced by phytoplankton and other sources in shallow eutrophic lakes (Wuhan, China): taxon-specific versus bulk activity. *Limnology* 10 95–104. 10.1007/s10201-009-0265-9

[B6] Cao X. Y. Štrojsová A. Znachor P. Zapomělová E. Liu G. X. Vrba J. (2005). Detection of extracellular phosphatases in natural spring phytoplankton of a shallow eutrophic lake (Donghu, China). *Eur. J. Phycol.* 40 251–258. 10.1080/09670260500192760

[B7] Carr O. J. Goulder R. (1990). Fish-farm effluents in rivers. I. Effects on bacterial populations an alkaline phosphatase activity. *Water Res.* 24 631–638. 10.1016/0043-1354(90)90196-D

[B8] Cembella A. D. Antia N. J. Harrison P. J. (1984). The utilization of inorganic and organic phosphorus compounds as nutrients by eukaryotic microalgae: a multidisciplinary perspective: Part I. *Crit. Rev. Microbiol.* 10 317–391. 10.3109/10408418209113567 6321101

[B9] Chróst R. J. (1991). “Environmental control of the synthesis and activity of aquatic microbial ectoenzymes,” in *Microbial Enzymes in Aquatic Environments* ed. Chróst R. J. (New York, NY: Springer-Verlag) 29–59.

[B10] Cotner J. B. Wetzel R. G. (1991). 5′-Nucleotidase activity in a eutrophic lake and an oligotrophic lake. *Appl. Environ. Microbiol.* 57 1306–1312. 16348478 10.1128/aem.57.5.1306-1312.1991 PMC182947

[B11] Cotner J. B. Wetzel R. G. (1992). Uptake of dissolved inorganic and organic phosphorus compounds by phytoplankton and bacterioplankton. *Limnol. Oceanogr.* 37 232–243. 10.4319/lo.1992.37.2.0232

[B12] Currie D. J. Kalff J. (1984). A comparison of the abilities of freshwater algae and bacteria to acquire and retain phosphorus. *Limnol. Oceanogr.* 29 298–310. 10.4319/lo.1984.29.2.0298

[B13] Dell Inc. (2016). *Dell Statistica (Data Analysis Software System), Version 13.2.*

[B14] Dignum M. Hoogveld H. L. Matthijs H. C. P. Laanbroek H. J. Pel R. (2004). Detecting the phosphate status of phytoplankton by enzyme-labelled fluorescence and flow cytometry. *FEMS Microbiol. Ecol.* 48 29–38. 10.1016/j.femsec.2003.12.007 19712428

[B15] Dyhrman S. T. Palenik B. (1999). Phosphate stress in cultures and field populations of the dinoflagellate *Prorocentrum minimum* detected by a single-cell alkaline phosphatase assay. *Appl. Environ. Microbiol.* 65 3205–3212. 10388722 10.1128/aem.65.7.3205-3212.1999 PMC91475

[B16] Dyhrman S. T. Ruttenberg K. C. (2006). Presence and regulation of alkaline phosphatase activity in eukaryotic phytoplankton from the coastal ocean: implications for dissolved organic phosphorus remineralization. *Limnol. Oceanogr.* 51 1381–1390. 10.4319/lo.2006.51.3.1381

[B17] González-Gil S. Keafer B. A. Jovine R. V. M. Aguilera A. Lu S. Anderson D. M. (1998). Detection and quantification of alkaline phosphatase in single cells of phosphorus-starved marine phytoplankton. *Mar. Ecol. Prog. Ser.* 164 21–35. 10.3354/meps164021

[B18] Healey F. P. Hendzel L. L. (1979). Fluorometric measurement of alkaline-phosphatase activity in algae. *Freshwat. Biol.* 9 429–439. 10.1111/j.1365-2427.1979.tb01527.x

[B19] Healey F. P. Hendzel L. L. (1980). Physiological indicators of nutrient deficiency in lake phytoplankton. *Can. J. Fish. Aquat. Sci.* 37 442–453. 10.1139/f80-058

[B20] Hoppe H. G. (1983). Significance of exoenzymatic activities in the ecology of brackish water: measurements by means of methylumbelliferyl substrates. *Mar. Ecol. Prog. Ser.* 11 299–308. 10.3354/meps011299

[B21] Hoppe H. G. (2003). Phosphatase activity in the sea. *Hydrobiologia* 493 187–200. 10.1023/A:1025453918247

[B22] Hrdinka T. Šobr M. Fott J. Nedbalová L. (2013). The unique environment of the most acidified permanently meromictic lake in the Czech Republic. *Limnologica* 43 417–426. 10.1016/j.limno.2013.01.005

[B23] Huang B. Q. Huang S. Y. Wen Y. Hong H. S. (2000). Effects of dissolved phosphorus on alkaline phosphatase activity in marine microalgae. *Acta Oceanol. Sin.* 19 29–35.

[B24] Huang Z. Terpetschnig E. You W. Haugland R. P. (1992). 2-(2′-phosphoryloxyphenyl)-4(3H)-quinazolinone derivates as fluorogenic precipitating substrates of phosphatases. *Anal. Biochem.* 207 32–39. 10.1016/0003-2697(92)90495-S 1336935

[B25] Jansson M. Olsson H. Pettersson K. (1988). Phosphatases; origin, characteristics and function in lakes. *Hydrobiologia* 170 157–175. 10.1007/BF00024903

[B26] Jones J. G. (1972). Studies on freshwater micro-organisms: phosphatase activity in lakes of differing degrees of eutrophication. *J. Ecol.* 60 777–791. 10.2307/2258564

[B27] Knoll L. B. Morgan A. Vanni M. J. Leach T. H. Williamson T. J. Brentrup J. A. (2016). Quantifying pelagic phosphorus regeneration using three methods in lakes of varying productivity. *Inland Waters* 6 509–522. 10.1080/IW-6.4.866

[B28] Litchman E. Nguyen B. L. V. (2008). Alkaline phosphatase activity as a function of internal phosphorus concentration in freshwater phytoplankton. *J. Phycol.* 44 1379–1383. 10.1111/j.1529-8817.2008.00598.x 27039852

[B29] Mindl B. Sonntag B. Pernthaler J. Vrba J. Psenner R. Posch T. (2005). Effects of phosphorus loading on the interactions of algae and bacteria: a reinvestigation of the “phytoplankton-bacteria paradox” in a continuous cultivation system. *Aquat. Microb. Ecol.* 38 203–213. 10.3354/ame038203

[B30] Nagata T. Kirchman D. L. (1992). Release of macromolecular organic complexes by heterotrophic marine flagellates. *Mar. Ecol. Prog. Ser.* 83 233–240. 10.3354/meps083233

[B31] Nedoma J. García J. Comerma M. Šimek K. Armengol J. (2006). Extracellular phosphatases in a Mediterranean reservoir: seasonal, spatial and kinetic heterogeneity. *Freshwat. Biol.* 51 1264–1276. 10.1111/j.1365-2427.2006.01566.x

[B32] Nedoma J. Padisák J. Koschel R. (2003a). Utilisation of 32P-labelled nucleotide- and non-nucleotide dissolved organic phosphorus by freshwater plankton. *Arch. Hydrobiol. Adv. Limnol.* 58 87–99.

[B33] Nedoma J. Porcalová A. Komárková J. Vyhnálek V. (1993). Phosphorus deficiency diagnostics in the eutrophic Římov reservoir. *Water Sci. Technol.* 28 75–84.

[B34] Nedoma J. Štrojsová A. Vrba J. Komárková J. Šimek K. (2003b). Extracellular phosphatase activity of natural plankton studied with ELF97 phosphate: fluorescence quantification and labelling kinetics. *Environ. Microbiol.* 5 462–472. 10.1046/j.1462-2920.2003.00431.x 12755713

[B35] Nedoma J. Vrba J. (2006). Specific activity of cell-surface acid phosphatase in different bacterioplankton morphotypes in an acidified mountain lake. *Environ. Microbiol.* 8 1271–1279. 10.1111/j.1462-2920.2006.01023.x 16817935

[B36] Novotná J. Nedbalová L. Kopáček J. Vrba J. (2010). Cell-specific extracellular phosphatase activity of dinoflagellate populations in acidified mountain lakes. *J. Phycol.* 46 635–644. 10.1111/j.1529-8817.2010.00858.x

[B37] Ou L. J. Huang B. Q. Hong H. S. Qi Y. Z. Lu S. H. (2010). Comparative alkaline phosphatase characteristics of the algal bloom dinoflagellates *Prorocentrum donghaiense* and *Alexandrium catenella*, and the diatom *Skeletonema costatum*. *J. Phycol.* 46 260–265. 10.1111/j.1529-8817.2009.00800.x

[B38] Ren L. X. Wang P. F. Wang C. Chen J. Hou J. Qian J. (2017). Algal growth and utilization of phosphorus studied by combined mono-culture and co-culture experiments. *Environ. Pollut.* 220 274–285. 10.1016/j.envpol.2016.09.061 27665120

[B39] Rengefors K. Pettersson K. Blenckner T. Anderson D. M. (2001). Species-specific alkaline phosphatase activity in freshwater spring phytoplankton: application of a novel method. *J. Plankton Res.* 23 435–443. 10.1093/plankt/23.4.435

[B40] Rengefors K. Ruttenberg K. C. Haupert C. L. Taylor C. Howes B. L. (2003). Experimental investigation of taxon-specific response of alkaline phosphatase activity in natural freshwater phytoplankton. *Limnol. Oceanogr.* 48 1167–1175. 10.4319/lo.2003.48.3.1167

[B41] Reynolds C. S. (1997). *Vegetation Processes in the Pelagic: A Model for Ecosystem Theory.* Oldenburg: Ecology Institute.

[B42] Rychtecký P. Řeháková K. Kozlíková E. Vrba J. (2015). Light availability may control extracellular phosphatase production in the turbid environment. *Microb. Ecol.* 69 37–44. 10.1007/s00248-014-0483-5 25190580

[B43] Schindler D. W. (2012). The dilemma of controlling cultural eutrophication of lakes. *Proc. Biol. Sci.* 279 4322–4333. 10.1098/rspb.2012.1032 22915669 PMC3479793

[B44] Schindler D. W. Carpenter S. R. Chapra S. C. Hecky R. E. Orihel D. M. (2016). Reducing phosphorus to curb lake eutrophication is a success. *Environ. Sci. Technol.* 50 8923–8929. 10.1021/acs.est.6b02204 27494041

[B45] Siuda W. Chróst R. J. (2001). Utilization of selected dissolved organic phosphorus compounds by bacteria in lake water under non-limiting orthophosphate conditions. *Pol. J. Environ. Stud.* 10 475–483.

[B46] Sommer U. (1981). The role of r- and K-selection in the succession of phytoplankton in Lake Constance. *Acta Oecol.* 2 327–342.

[B47] Sommer U. (1985). Comparison between steady state and non-steady state competition: experiments with natural phytoplankton. *Limnol. Oceanogr.* 30 335–346. 10.4319/lo.1985.30.2.0335

[B48] Štrojsová A. Nedoma J. Štrojsová M. Cao X. Vrba J. (2008). The role of cell-surface-bound phosphatases in species competition within natural phytoplankton assemblage: an *in situ* experiment. *J. Limnol.* 67 128–138. 10.4081/jlimnol.2008.128

[B49] Štrojsová A. Vrba J. (2006). Phytoplankton extracellular phosphatases: investigation using ELF (Enzyme Labelled Fluorescence) technique. *Pol. J. Ecol.* 54 715–723.

[B50] Štrojsová A. Vrba J. (2009). Short-term variation in extracellular phosphatase activity: possible limitations for diagnosis of nutrient status in particular algal populations. *Aquat. Ecol.* 43 19–25. 10.1007/S10452-007-9154-7

[B51] Štrojsová A. Vrba J. Nedoma J. Komárková J. Znachor P. (2003). Seasonal study on expression of extracellular phosphatases in the phytoplankton of an eutrophic reservoir. *Eur. J. Phycol.* 38 295–306.

[B52] Štrojsová A. Vrba J. Nedoma J. Šimek K. (2005). Extracellular phosphatase activity of freshwater phytoplankton exposed in different *in situ* phosphorus concentrations. *Mar. Freshw. Res.* 56 417–424. 10.1071/MF04283

[B53] Vrba J. Komárková J. Vyhnálek V. (1993). Enhanced activity of alkaline phosphatases – phytoplankton response to epilimnetic phosphorus depletion. *Water Sci. Technol.* 28 15–24.

[B54] Vrba J. Kopáček J. Bittl T. Nedoma J. Štrojsová A. Nedbalová L. (2006). A key role of aluminium in phosphorus availability, food web structure, and plankton dynamics in strongly acidified lakes. *Biologia* 61 S441–S451. 10.2478/s11756-007-0077-5

[B55] Vrba J. Kopáček J. Fott J. Kohout L. Nedbalová L. Pračáková M. (2003). Long-term studies (1871–2000) on acidification and recovery of lakes in the Bohemian Forest (central Europe). *Sci. Total Environ.* 310 73–85. 10.1016/S0048-9697(02)00624-1 12812732

[B56] Wetzel R. G. (1991). “Extracellular enzymatic interactions: storage, redistribution, and interspecific communication,” in *Microbial Enzymes in Aquatic Environments* ed. Chróst R. J. (New York, NY: Springer-Verlag) 6–28.

[B57] Young E. B. Tucker R. C. Pansch L. A. (2010). Alkaline phosphatase in freshwater *Cladophora*-epiphyte assemblages: regulation in response to phosphorus supply and localization. *J. Phycol.* 46 93–101. 10.1111/j.1529-8817.2009.00782.x

